# Unexpected Diversity of Chloroplast Noncoding RNAs as Revealed by Deep Sequencing of the *Arabidopsis* Transcriptome

**DOI:** 10.1534/g3.111.000752

**Published:** 2011-12-01

**Authors:** Amber M. Hotto, Robert J. Schmitz, Zhangjun Fei, Joseph R. Ecker, David B. Stern

**Affiliations:** * Boyce Thompson Institute for Plant Research and; ‡ Robert W. Holley Center for Agriculture and Health, Agricultural Research Service, United States Department of Agriculture, Ithaca, New York 14853; † Plant Biology and Genomic Analysis Laboratorys; § Howard Hughes Medical Institute, The Salk Institute for Biological Studies, La Jolla, California 92037

**Keywords:** RNA-Seq, posttranscriptional regulation, transcription, organelle, plastid

## Abstract

Noncoding RNAs (ncRNA) are widely expressed in both prokaryotes and eukaryotes. Eukaryotic ncRNAs are commonly micro- and small-interfering RNAs (18–25 nt) involved in posttranscriptional gene silencing, whereas prokaryotic ncRNAs vary in size and are involved in various aspects of gene regulation. Given the prokaryotic origin of organelles, the presence of ncRNAs might be expected; however, the full spectrum of organellar ncRNAs has not been determined systematically. Here, strand-specific RNA-Seq analysis was used to identify 107 candidate ncRNAs from *Arabidopsis thaliana* chloroplasts, primarily encoded opposite protein-coding and tRNA genes. Forty-eight ncRNAs were shown to accumulate by RNA gel blot as discrete transcripts in wild-type (WT) plants and/or the *pnp1-1* mutant, which lacks the chloroplast ribonuclease polynucleotide phosphorylase (cpPNPase). Ninety-eight percent of the ncRNAs detected by RNA gel blot had different transcript patterns between WT and *pnp1-1*, suggesting cpPNPase has a significant role in chloroplast ncRNA biogenesis and accumulation. Analysis of materials deficient for other major chloroplast ribonucleases, RNase R, RNase E, and RNase J, showed differential effects on ncRNA accumulation and/or form, suggesting specificity in RNase-ncRNA interactions. 5′ end mapping demonstrates that some ncRNAs are transcribed from dedicated promoters, whereas others result from transcriptional read-through. Finally, correlations between accumulation of some ncRNAs and the symmetrically transcribed sense RNA are consistent with a role in RNA stability. Overall, our data suggest that this extensive population of ncRNAs has the potential to underpin a previously underappreciated regulatory mode in the chloroplast.

Both prokaryotes and eukaryotes express a large number of noncoding RNAs (ncRNA) antisense to coding regions, ranging from ∼9% (*Arabidopsis*) to 29% (mouse) of identified genes (Jen *et al.* 2005; Wang *et al.* 2005; Zhang *et al.* 2006). Although the term ncRNA refers to abundant transcripts, such as rRNAs and tRNAs, it has more recently incorporated regulatory RNAs, such as bacterial small RNAs (sRNA; <400 nt), eukaryotic micro- and small-interfering RNAs (miRNA and siRNA), antisense RNAs (asRNA) and long noncoding RNAs (lncRNA). The present study focuses on asRNAs, which can be divided into two groups based on the target interaction: *cis*-encoded asRNAs bind to and regulate the complementary sense RNA, and *trans*-encoded asRNAs act on one or more unlinked loci through short regions of complementarity. Base pairing of these asRNAs to their targets can elicit translational inactivation/activation, mRNA stabilization/destabilization, or differential transcription termination (Storz *et al.* 2005; Repoila and Darfeuille 2009).

The occurrence of plant organellar ncRNAs has been established by limited analysis of specific cDNA populations. Studies have revealed mitochondrial ncRNAs from wild-type (WT) and mitochondrial polynucleotide phosphorylase (PNPase)–deficient *Arabidopsis* (Holec *et al.* 2006; Lung *et al.* 2006) and short (<500 nt) ncRNAs from tobacco and *Arabidopsis* chloroplasts (Marker *et al.* 2002; Lung *et al.* 2006). Subsequent studies have attempted to elucidate the regulatory roles of chloroplast ncRNAs. In one case, an *ndhB* asRNA was hypothesized to stabilize or regulate maturation of the cognate sense transcript, whereas a *psbT* asRNA was proposed to regulate accumulation of PsbT protein through occlusion of the *psbT* ribosomal binding site (Georg *et al.* 2010; Zghidi-Abouzid *et al.* 2011). Our own work suggested a role for an asRNA complementary to the 5S rRNA, AS5, in regulating the processing and accumulation of 5S rRNA (Hotto *et al.* 2010; Sharwood *et al.* 2011).

There are at least two indications that the organellar ncRNA population might be significantly more complex than elucidated to date. First, in cyanobacteria, which represent the chloroplast progenitor, numerous ncRNAs have been identified, some of which accumulate differentially in response to stress or developmental stage (Steglich *et al.* 2008; Georg *et al.* 2009). Second, transcription termination in chloroplasts has long been known to be inefficient (Stern and Gruissem 1987), suggesting that intergenic and antisense regions may be readily transcribed.

Taken together, it is clear that ncRNAs accumulate in chloroplasts, and available evidence favors functional roles in gene expression, at least for some. To gain a more complete picture of this population, we have used strand-specific, high-throughput RNA sequencing (RNA-Seq) of total RNA. We present data extracted from this sequencing, demonstrating that accumulating ncRNAs are derived from much of the chloroplast genome. The biogenesis and regulation of these RNAs are further examined through analysis of chloroplast ribonuclease mutants and 5′ end mapping.

## Materials and Methods

### Plant growth conditions and material

*Arabidopsis thaliana* Columbia ecotype (Col-0) was used as the WT for this study. The three T-DNA mutants used contain insertions in the genes At3g03710 (*pnp1-1*; SALK_013306), At5g02250 (*rnr1-3*; SALK_090294), and At2g04270 (*rne1-1*; SALK_093546) and have been previously characterized (Alonso *et al.* 2003; Bollenbach *et al.* 2005; Mudd *et al.* 2008; Marchive *et al.* 2009). WT and *pnp1-1* plants were germinated and grown on soil with a 16-h light/dark photoperiod (150 μmol m^-2^ s^-1^ intensity). The *rnr1-3*, *rne1-1*, and WT seeds were surface sterilized and germinated on MS-agar medium under 100 μmol m^-2^ s^-1^ light with a 16-h light/dark photoperiod. At 25 days postgermination, *rne1-1* plants and the WT control were transferred to soil and grown under 150 μmol m^-2^ s^-1^ light. All plants were grown at 25°. Leaf tissue was harvested after 40 days from *rne1-1*, *rnr1-3*, and WT and after 25 days for soil-grown *pnp1-1* and WT, frozen in liquid nitrogen, and stored at −80° for subsequent analysis. RNase J–deficient material was produced using virus-induced gene silencing (VIGS) as previously described (Sharwood *et al.* 2011). Affected tissue and empty vector control samples were harvested, frozen in liquid nitrogen, and stored at −80°.

Plants deficient for the plastid-encoded RNA polymerase (PEP) were generated by germination on MS-agar medium containing 500 μg mL^-1^ spectinomycin dihydrochloride pentahydrate (Sigma-Aldrich) (Swiatecka-Hagenbruch *et al.* 2007). After stratification, plants were placed under an 8-h light/dark photoperiod for 14 days, then transferred to a 16-h light/dark photoperiod for an additional 7 days (150 μmol m^-2^ s^-1^ light intensity) prior to harvest.

### RNA-Seq

Total RNA was extracted using the RNeasy Plant Mini Kit (Qiagen) from two WT and two *pnp1-1* samples. Ten micrograms total RNA were depleted of rRNAs using the plant RiboMinus-Kit (Invitrogen). Strand-specific RNA-Seq libraries were prepared following the “Directional mRNA-Seq Library Prep Pre-Release” protocol by Illumina. Each sample was sequenced for 85 cycles on an Illumina GAIIx. Image analysis and base calling were performed with the standard Illumina pipeline (Firecrest v1.3.4 and Bustard v.1.3.4). The resulting reads were aligned to the *Arabidopsis* genome (TAIR9) using Tophat (version 1.0.13)/Bowtie (version 0.12.3) with the following commands: -F 0 -g 2 -I 5000 (Langmead *et al.* 2009; Trapnell *et al.* 2010). Up to two locations were accepted for placement of sequenced reads to allow mapping to the large inverted repeat of the chloroplast genome. Sequence data can be downloaded from National Center for Biotechnology Information Sequence Read Archive SRA046998.

### RNA isolation, RT-PCR, and RNA gel blots

Mature leaf tissue was ground in liquid nitrogen, and total RNA extracted using TRI reagent (Molecular Research Center) with minor modifications to the manufacturer’s instructions. RNA was precipitated overnight with isopropanol at −20°, and the pellet was washed with 75% ethanol and dissolved in water. Primers were designed for cDNA synthesis, PCR, and RNA blot probe synthesis to amplify a ≤100 nt ncRNA section as determined from the RNA-Seq data using Primer3 (Rozen and Skaletsky 2000). For strand-specific cDNA synthesis, 1 μg of DNase-treated RNA (Promega) was reverse-transcribed with SuperScript III (Invitrogen) using 2 μM of the 3′ ncRNA gene-specific primers (supporting information, Table S1). The PCR reaction contained 1X Master Mix, 0.2 mM each dNTP, 400 nM each 5′ and 3′ primer, 1.25 U GoTaq DNA polymerase (Promega), and 100 ng of cDNA in a 25 μL reaction volume. Amplification was completed with the following protocol: initial denaturation at 94° for 3 min, then 30 cycles at 94° for 30 s, 60° for 30 s, and 72° for 30 s, and a final extension of 72° for 7 min. Amplicons were visualized after migration in 2% agarose gels.

For RNA gel blot analysis, 5 μg of total RNA was separated in 1.2% agarose/formaldehyde gels, which were blotted overnight onto Hybond-N+ (GE Healthcare) in 25 mM sodium phosphate buffer. Membranes were probed with single-stranded RNA or double-stranded DNA probes as indicated in the figure legends. The ncRNA templates for probe synthesis were amplified with the 5′ primer and the 3′ primer containing a T7 promoter, and the sense strand templates for probe synthesis were amplified with the corresponding ncRNA 3′ primer and 5′ primer containing a T7 promoter. The only exception was the sense strand tRNA template, which was amplified with tRNA-specific primers given in Table S1. RNA probes were made from 100 ng of template using T7 RNA polymerase (Promega) and 40 μCi α-^32^P-UTP, and purification through a Sephadex G-25 column. Membrane hybridization and washing were performed as previously described (Hotto *et al.* 2010). Where indicated in Figure 4, dsDNA probes synthesized from 100 ng of template were used, with hybridization according to Church and Gilbert (1984).

### 5′ RACE

5′ RACE used the GeneRacer Kit (Invitrogen) with minor modifications. DNase-treated (Ambion) total RNA (4 μg) was incubated with and without tobacco acid phosphatase (TAP), followed by ligation to the GeneRacer RNA Oligo with T4 RNA ligase (treatment with calf intestinal phosphatase was omitted). The ncRNA cDNA was synthesized with SuperScript III using 3′ gene-specific primers (Table S1). The 5′ cDNA ends were amplified by PCR using 0.02 U μL^-1^ Phusion high-fidelity DNA polymerase (Thermo Scientific), 0.9 μM GeneRacer 5′ primer, 0.3 μM 3′ gene-specific primer, 1X Phusion HF buffer, 300 nM each dNTP, and 8 ng of cDNA in a 50 μL reaction volume. The following cycling protocol was used: initial denaturation at 98° for 30 s, then 35 cycles at 98° for 10 s, 64° for 20 s, and 72° for 60 s, and a final extension at 72° for 5 min. Nested PCR was performed using 1 μl of the initial PCR as a template with 0.2 μM each 3′ nested primer and GeneRacer 5′ nested primer, 200 nM each dNTP, and either 1X Phusion HF buffer with 0.02 U μL^-1^ Phusion DNA polymerase or 1X GoTaq buffer with 1.25 U GoTaq DNA polymerase in a 50 μL reaction volume. The cycling protocol used for nested PCR with Phusion polymerase was the same as above, except the annealing temperature was 65°. The cycling protocol for nested PCR with GoTaq polymerase was as follows: initial denaturation at 94° for 3 min, then 35 cycles at 94° for 30 s, 60° for 30 s, and 72° for 90 s, and a final extension at 72° for 5 min. 5′ RACE products were visualized in 1% agarose gels. Specific bands were gel extracted using the Wizard SV Gel Clean-up System (Promega) and either directly sequenced or cloned into pCR 4-TOPO and then sequenced. Sequences were aligned to the *Arabidopsis* chloroplast genome (accession number NC_000932).

## Results

### Analysis of the *Arabidopsis* chloroplast transcriptome reveals 107 ncRNA candidates

Total RNA isolated from mature leaves of *Arabidopsis* WT and the chloroplast PNPase (cpPNPase) null mutant *pnp1-1* was sequenced to identify potential nc- and asRNAs. We examined *pnp1-1* because PNPase has an active role in bacterial ncRNA regulation, and *Arabidopsis* mitochondrial ncRNAs overaccumulate in plants deficient for the mitochondrial isozyme (Holec *et al.* 2006; Viegas *et al.* 2007; Bollenbach *et al.* 2008). To achieve strand-specific sequencing of the chloroplast transcriptome, 5′ and 3′ oligonucleotides were sequentially added to total RNA after depletion of rRNA and metal hydrolysis, a method that retains transcripts longer than approximately 80 nt. The Illumina Genome Analyzer IIx platform was used to sequence this pool, resulting in a strand-specific RNA-Seq dataset. Sequences that aligned to the chloroplast genome were extracted, resulting in an average of 10,545,033 reads per sample and coverage of greater than 99% of both strands of the WT chloroplast genome, including unannotated regions. For this study, we focused on identification of putative asRNAs with at least partial complementarity to a known sense transcript and identification of ncRNAs that are complementary to intergenic regions within known gene clusters, thus excluding possible ncRNAs derived from cleavage of known functional transcripts. To limit identification of false positives, a minimum of 50× coverage at any nucleotide in the WT and/or *pnp1-1* was defined as a transcription peak corresponding to an ncRNA candidate.

**Figure 1:**
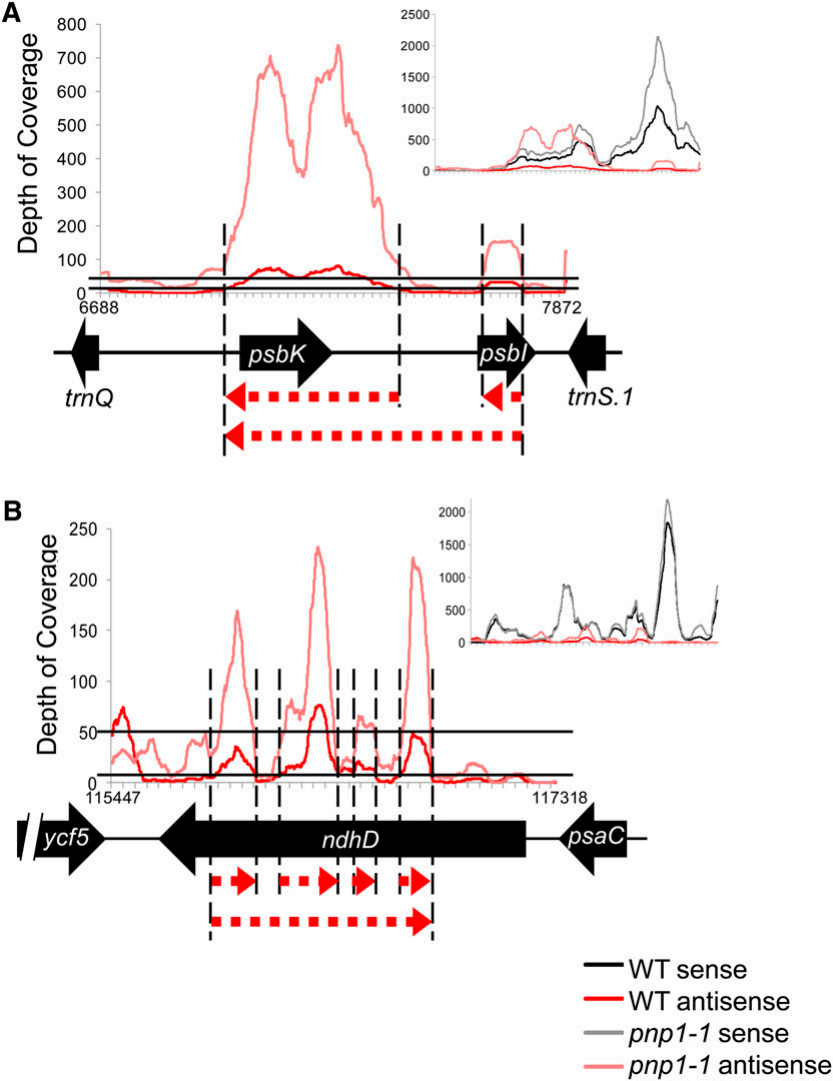
Identification of *Arabidopsis* chloroplast ncRNAs from RNA-Seq data. Graphs represent the depth of coverage of ncRNA *vs.* genome position from 6688–7872 bp (A) or 115447–117318 bp (B). The insets contain both sense (black/gray) and antisense (red/pink) reads. Below each graph is the gene model for the area, including known genes (black arrows) and potential ncRNAs (dashed red arrows). Vertical dashed lines correspond to potential 5′ and 3′ ends of ncRNAs, based on the RNA-Seq peaks in WT. The solid horizontal lines delineate the thresholds of 10 and 50 reads, as discussed in the text.

One challenge was identifying the 5′ and 3′ ends of novel transcripts, particularly those of low abundance. Since RNA-Seq relies on ligation of oligonucleotides to the RNA ends, those ends engaged in secondary structures will be underrepresented, resulting in an uneven distribution of reads across contiguous transcripts and low coverage of ends (Wang *et al.* 2009; Roberts *et al.* 2011). For the purpose of this study, an end was assigned when the sequencing coverage fell below 10×. As an example of the application of these criteria, Figure 1A shows two candidate asRNAs, as-*psbK* and as-*psbI*, and Figure 1B shows four potential asRNAs, as-*ndhD*1–4. In these cases, the occurrence of an asRNA candidate was prompted when the depth of coverage exceeded 50× at a single nucleotide, and it was assumed to continue in both directions until the coverage dropped below 10×. In Figure 1A, this resulted in the identification of two asRNAs in this region with a gap between them, as annotated in Table 1. Under such circumstances, however, two peaks might actually correspond to a single ncRNA with poor sequence coverage in the intervening region, as shown by the continuous dashed red line. The *ndhD* situation was more complex, given that the four individual asRNA peaks might represent as few as one, or as many as four, actual ncRNA species. This type of ambiguity arose rather frequently, with the extreme example being the 10 transcripts defined as antisense to *ycf2.1*. The same phenomenon was also seen when sense strand transcripts were examined (insets in Figure 1). Therefore, the fragmentation of transcripts appears to be a limitation to the RNA-Seq method, rather than a peculiarity of ncRNAs themselves.

**Table 1:** Noncoding RNAs predicted by RNA-Seq data

						RNA Blot Estimated Band Sizes (kb)	
ncRNA designation	Antisense Gene*^a^*	Start*^b^*	Stop*^b^*	Strand*^c^*	RT-PCR	WT	*pnp1-1*
1	*trnH*	18	125	+	+	1.6, 1.8	1.6, 1.8, 3.7
2	*matK*	2946	3086	+	+	—	—
3	*rps16*	4814	5371	+	+	1.3	0.8, 1.2,1.3
4	*rps16*i-1	5426	5581	+	+	0.8, 1.1,1.3	0.8, 0.9, 1.1,1.3
5	rps16i-2	5632	5875	+	NT	NT	NT
6	*trnQ*	6521	6646	+	+	1.0, 1.2	1.1, 1.2
7	*psbK*	6882	7462	−	+	1.2, 3.3, 3.4	1.3, 3.4, 3.5
8	*psbI*	7611	7691	−	−	NT	NT
9	*trnS.1*	7817	7984	+	+	1.8, 3.0, 3.2	1.9, 3.1, 3.3
10	*trnG*-5′	8644	9004	−	+	1.1, 1.8	1.1, 1.8, 3.7
11	*trnG*-3′	9244	9484	−	+	—	0.5, 0.6, 1.0, 3.3, 3.7
12	*trnR.1*	9555	9858	−	+	0.2, 0.3	—
13	*atpA*	10342	10536	−	NT	NT	NT
14	*atpI*	14088	14182	+	+	0.6, 1.0	0.2, 0.4, 0.6, 1.0, 1.2
15	*rpoC2*-1	16984	17163	+	NT	NT	NT
16	*rpoC2*-2	17233	17410	+	NT	NT	NT
17	*trnC*	27446	27666	−	+	0.6, 1.1, 1.7	0.5, 0.6, 1.1, 1.7
18	*trnC-ycf6*int	27807	27956	−	+	—	—
19	*trnD*	29627	29825	+	NT	NT	NT
20	*trnD-trnY*int	29939	30070	+	NT	NT	NT
21	*trnT.1*	31408	31518	−	+	—	0.3, 0.9
22	*psbD*	33106	33456	−	+	—	—
23	*trnS.2*	35312	35409	+	+	3.3, 3.4	3.1, 3.4, 3.7
24	*ycf9*	35820	36116	−	+	0.3, 0.9, 2.0	0.4, 0.8, 1.0, 2.3
25	*trnfM*	36642	36862	+	+	0.1, 0.3, 0.4	0.8-1.0 smear
26	*rps14-psaB*int	37250	37327	+	−	NT	NT
27	*ycf3*-3′	42542	42629	+	+	—	—
28	*trnS.3*	44827	45050	−	+	1.1	1.2, 1.4, 1.5, 3.0
29	*rps4*-3′	45294	45358	+	+	1.9	1.9
30	*rps4*-5′	45747	45929	+	−	—	—
31	*trnT.2*	46026	46338	+	−	—	—
32	*trnL*-3′	47417	47670	−	NT	NT	NT
33	*ndhK*	49260	49386	+	+	—	—
34	*ndhC*-5′	50269	50469	+	+	—	—
35	*ndhC-trnV.1*int	50762	50916	+	+	—	0.4, 0.7, 0.8, 1.3
36	*trnV*i	51688	51884	+	NT	NT	NT
37	*trnM*	51950	52128	−	NT	NT	NT
38	*rbcL-accD*int	56584	56822	−	+	0.5, 0.6, 1.0	0.4, 0.5, 3.0
39	*accD*	57949	58056	−	+	0.7, 0.8, 1.0, 1.9	0.4, 0.7, 1.9
40	*accD-psaI*int-1	58776	58869	−	+	1.1, 1.8	0.4, 0.6, 0.7, 1.1, 1.9
41	*accD-psaI*int-2	59030	59096	−	−	NT	NT
42	*ycf10*-5′	60756	60963	−	+	—	—
43	*ycf10*	60983	61159	−	+	1.3, 1.7, 1.8	1.1, 1.3, 1.7, 1.9
44	*ycf10-petA*int	61409	61529	−	+	1.0, 1.6, 1.8	0.9, 1.0, 1.6, 1.8
45	*petA*	62045	62271	−	+	0.5, 1.7	0.5, 1.2, 1.4, 1.8
46	*psbJ*	63479	63648	+	NT	NT	NT
47	*psbL-psbF*	63805	64018	+	NT	NT	NT
48	*orf31*	65708	65805	−	+	—	—
49	*trnW*	66248	66330	+	+	0.9, 1.3, 1.8	0.8, 0.9, 1.2, 1.3, 1.9, 2.9
50	*trnP*	66330	66701	+	+	1.0, 1.1	1.0, 1.1, 1.9
51	*psaJ*	66920	67113	−	NT	NT	NT
52	*psaJ-rpl33*int	67154	67304	−	+	0.3, 0.7, 1.3, 1.4	1.2, 1.3, 1.4, 3.0
53	*rpl33*	67334	67594	−	+	0.7, 1.3, 1.4, 2.7, 3.1	1.2, 1.3, 1.4, 3.0
54	*rps18*	67784	68222	−	+	1.0, 1.4, 1.9, 3.0	1.2, 1.8, 1.9, 2.8, 3.7
55	*rpl20*	68512	68680	+	NT	NT	NT
56	*psbB*	73677	73837	−	+	1.7	0.9, 1.0, 1.7
57	*psbB-psbT*int	73927	74077	−	+	0.6	0.6, 0.7, 0.9
58	*psbT*	74136	74184	−	NT	NT	NT
59	*psbN*	74249	74380	+	+	2.0, 3.3, 3.5	2.0, 3.3, 3.6
60	*psbH*	74601	74726	−	+	1.1, 1.9	1.9
61	*petB*i	74967	75151	−	+	—	—
62	*petD*-3′	77431	77604	−	+	—	—
63	*rpoA*-3′	77967	78098	+	+	—	1.2, 1.3, 3.3
64	*rpoA*	78320	78768	+	NT	NT	NT
65	*rpoA*-5′	78827	78957	+	+	—	—
66	*rps11*	79166	79359	+	NT	NT	NT
67	*rpl36*	79502	79858	+	NT	NT	NT
68	*rpl14*	80723	80895	+	NT	NT	NT
69	*rpl2*i	85271	85419	+	NT	NT	NT
70	*trnI.1*	86203	86389	+	+	0.3	0.35, 0.4, 0.5
71	*ycf2.1*-1	86644	86764	−	+	0.7	0.75
72	*ycf2.1*-2	87948	88212	−	+	—	1.2, 1.3, 3.5
73	*ycf2.1*-3	88451	88564	−	+	—	—
74	*ycf2.1*-4	88960	89328	−	+	—	—
75	*ycf2.1*-5	89445	90769	−	+	1.0	—
76	*ycf2.1*-6	91158	91564	−	+	—	1.2, 1.7
77	*ycf2.1*-7	91703	92005	−	+	—	—
78	*ycf2.1*-8	92309	92432	−	+	1.8, 3.7	1.1, 1.2, 1.8, 3.8
79	*ycf2.1*-9	92979	93113	−	+	1.2	0.6, 0.7, 1.25, 1.6, 1.7
80	*ycf2.1*-10	93178	93281	−	+	—	—
81	*trnL-ndhB*int	94377	94967	+	+	0.4, 1.7, 3.7	0.6, 1.6, 1.7, 3.7
82	*ndhB*-3′	95111	95707	+	+	—	—
83	*ndhB*i	95910	96164	+	NT	NT	NT
84	*rps12*-5′	98718	98822	+	NT	NT	NT
85	*rrn*4.5-*rrn5*int	107905	107963	−	+	—	—
86	*ycf1.1*-1	109354	109553	−	NT	NT	NT
87	*ycf1.1*-2	109923	110387	−	+	0.8, 0.9, 1.3, 3.3	0.85, 1.3, 3.2, 3.3
88	*ndhF*-3′	110389	110488	+	−	NT	NT
89	*ndhF*-5′-1	112345	112435	+	+	1.7, 1.8, 3.8	1.7, 1.8, 3.8
90	*ndhF*-5′-2	112475	112665	+	+	1.65, 1.7	1.75, 1.8
91	*ycf5*-1	114738	114982	−	NT	NT	NT
92	*ycf5*-2	115138	115220	−	+	—	—
93	*ndhD*-1	115897	116043	+	+	1.6	0.3, 1.3, 1.8
94	*ndhD*-2	116137	116437	+	+	1.3	1.35
95	*ndhD*-3	116467	116567	+	+	—	—
96	*ndhD*-4	116627	116785	+	−	NT	NT
97	*ndhA*-3′	119774	119911	+	NT	NT	NT
98	*ndhA*i	120852	120948	+	+	—	0.3, 0.5
99	*ndhA*i-2	121194	121344	+	NT	NT	NT
100	*ndhA*-5′	121398	121822	+	NT	NT	NT
101	*ycf1.2*-1	124129	124265	+	NT	NT	NT
102	*ycf1.2*-2	124605	124768	+	NT	NT	NT
103	*ycf1.2*-3	125263	125448	+	NT	NT	NT
104	*ycf1.2*-4	127146	127277	+	+	2.0	0.8, 2.0, 3.7
105	*ycf1.2*-5	127626	127788	+	+	—	—
106	*ycf1.2*-6	127788	128098	+	−	NT	NT
107	*ycf1.2*-7	128220	129004	+	NT	NT	NT

NT, not tested.

a Gene or genes encoded on complementary strand. 5′ or 3′ indicates which part of the coding region is complementary to the ncRNA. Some ncRNAs are between two coding regions (int) or opposite an intron (i).

b ncRNA termini as predicted by RNA-Seq from WT, except for the NT samples, which were defined from *pnp1-1* RNA-Seq data based on the criteria given in the text.

c Strand is + or – for the ncRNA according to the GenBank accession for *Arabidopsis* cpDNA.

When the RNA-Seq datasets from both genotypes were analyzed using these criteria, 107 ncRNA candidates were identified (Table 1), ranging in size from 48 to 1,300 nt, with an average size of 217 nt. Of this pool, 12 candidates were defined strictly as ncRNAs because they were complementary to intergenic regions within operons, whereas the remaining 95 candidates were asRNAs as they were antisense to known coding regions. Of the 107 candidates, 29 exceeded the 50× coverage only in *pnp1-1*, although most were also visible as peaks in the WT reads (*e.g.* as-*psbI* and as-*ndhD*1; Figure 1). Additionally, both sense and antisense transcript abundances were estimated by binning reads according to their start position against the TAIR9 genome annotation. Overall, asRNAs contained ∼4-fold more sequencing reads in *pnp1-1* compared with the WT (Figure 1 and Table S2). This observation explains, at least in part, why some candidates were identified from the mutant. It also suggests that, as in *Arabidopsis* mitochondria (Holec *et al.* 2006), cpPNPase may globally modulate ncRNA abundance.

**Figure 2:**
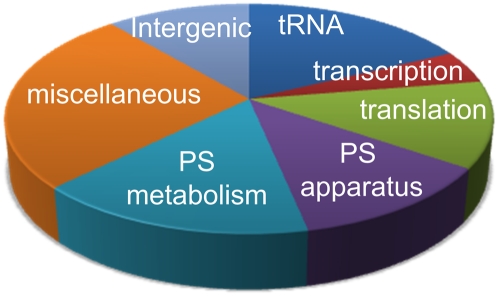
The proportion of ncRNAs antisense to sense strand coding region categories, as discussed in the text. PS, photosynthetic.

The 95 asRNA candidates mentioned above can be classified by the function of the complementary strand. As shown in Figure 2, the distribution of sense strand gene function was broad, with the largest group being miscellaneous protein-coding genes. These include several *ycf* (hypothetical coding frame) genes, which are genes of unknown function that are conserved between species. However, all gene classes were well represented except rRNAs. In total, more than half of the annotated chloroplast genes had one or more predicted asRNA counterparts. Putative locations of sense-antisense RNA pairing can also be used to predict function, including 5′ (∼31 asRNAs) and 3′ end pairing (∼39 asRNAs), and pairing within the coding region (∼40 asRNAs; see *Discussion*). Overall, this suggests a variety of possible regulatory functions and targets.

### ncRNA size and abundance differs between WT and *pnp1-1*

To explore whether the ncRNAs identified using RNA-Seq were discrete species, expression was first tested by RT-PCR. PCR primers were designed to amplify a ≤100 nt transcript segment toward the middle of the expression peak defined by RNA-Seq. Of the 107 ncRNA candidates, 77 were tested, and of these, 69 were detected by RT-PCR in the WT (Table 1). For the 8 that were negative, the most likely explanation is low transcript abundance; however, we did not repeat the analysis using alternative primer sets or RNA from *pnp1-1*.

**Figure 3:**
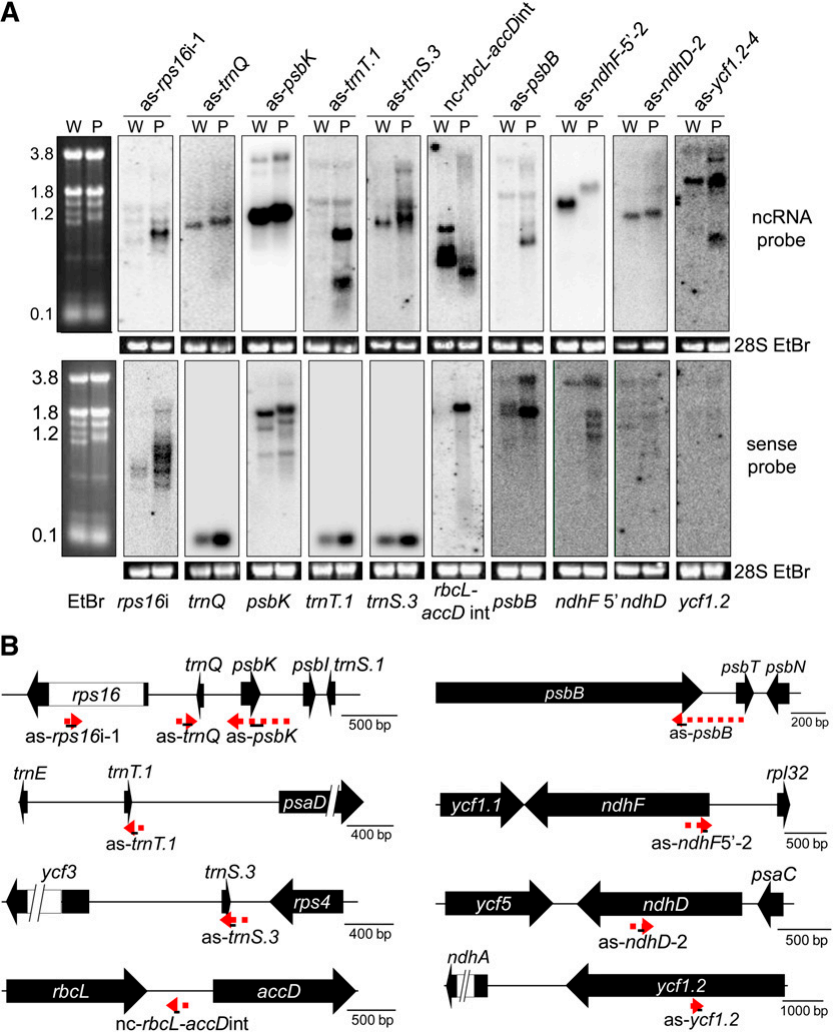
Analysis of 10 genic areas for nc- and sense RNAs. (A) RNA gel blot detection of ncRNAs (top) and the corresponding sense strand (bottom). Five micrograms of total RNA from wild-type (W) and *pnp1-1* (P) samples was separated in a 1.2% agarose-formaldehyde gel, blotted, and incubated with strand-specific probes. An ethidium bromide (EtBr)–stained gel is shown with rRNA sizes (kb; left). The 28S rRNA ethidium bromide–stained gel is shown below each blot to reflect loading. (B) Representative gene models for the nc- (red dashed arrows) and sense (filled black arrows) RNAs from (A). Solid black lines under the red arrows represent probes used for RNA blot analysis and amplicons for RT-PCR verification. Gene models are drawn to the scales shown at right.

Each of the 69 ncRNAs confirmed by RT-PCR was analyzed by RNA gel blot using WT and *pnp1-1* samples. Of these, 48 ncRNAs gave visible signals for the WT, *pnp1-1*, or both; however, the number, size, or abundance of transcripts often differed between WT and *pnp1-1* (Figure 3A and Figure S1). Transcript sizes ranged from ∼100 nt in WT and ∼200 nt in *pnp1-1* to >3 kb in both. In all but five cases, the transcript size was underestimated by the RNA-Seq data for all transcripts observable by RNA blot in both WT and *pnp1-1*. In one case, RNA-Seq overestimated the transcript size (as-*ycf2.1*-5, detected in WT only; Figure S1); in two cases, there was a transcript of approximately the predicted size in WT (as-*ycf9*, as-*trnR.1*); and in two cases, the transcript was smaller than the predicted transcript in WT (as-*trnfM*, nc-*trnL-ndhB*int).

The representative examples in Figure 3A show that transcript abundance, as estimated by hybridization signal intensity, ranged from low (*e.g.* as-*psbB*) to high (*e.g.* as-*psbK* and as-*ndhF*-5′-2). Additionally, the transcript patterns varied from simple (*e.g.* as-*psbK* and as-*ndhD*-2) to more complex (*e.g.* as-*ycf1.2*-4), suggestive of varying biogenesis pathways. Additional examples are shown in Figure S1. For the 21 ncRNAs not detected by gel blot (Table 1), we presume that they are of even lower abundance than species such as as-*psbB*.

The differences in ncRNA patterns between the WT and *pnp1-1* were in some instances quite dramatic. For example, some ncRNAs were only detectable in the *pnp1-1* sample (*e.g.* as-*rps16*i-1 and as-*psbB*), although they were originally verified by RT-PCR from WT. This suggests that their abundance is normally strongly limited by cpPNPase activity and that, in effect, *pnp1-1* is an overexpressor of these species, a finding consistent with RNA-Seq data (Table S2). In other cases, such as nc-*rbcL-accD* and as-*ycf1.2*-4, there were additional, shorter transcripts in *pnp1-1*. These could be degradation intermediates that persist in the absence of cpPNPase activity. Many ncRNAs were slightly longer in *pnp1-1* compared with the WT (*e.g.* as-*trnQ* and as-*psbK*). Because of the known functions of cpPNPase, the longer transcripts presumably include 3′ extensions. Taken together, these data point to cpPNPase as a key player for ncRNA processing and accumulation.

### Differential accumulation of sense-strand RNAs corresponding with altered ncRNA patterns in WT *vs.* *pnp1-1*

Because ncRNA transcript abundance and form differed between WT and *pnp1-1*, we decided to examine the corresponding sense strand transcripts for possible correlations between sense-antisense pairs for the 10 examples shown in Figure 3. In 7 of these 10 cases, the genotypes varied in ncRNA abundance and/or transcript number. In other cases, *pnp1-1* ncRNAs appeared to have 3′ extensions but similar accumulation. To facilitate the comparison, single-stranded probes for the sense and antisense strands were created from identical regions of the genome, except for tRNAs where the complete tRNA sequence was used regardless of the antisense probe location.

The three tRNA probes revealed an estimated 2- to 3-fold increased tRNA abundance in *pnp1-1* compared with the WT. This result is consistent with an earlier report implicating cpPNPase in the regulation of tRNAs (Walter *et al.* 2002). Interestingly, the abundance of the corresponding antisense tRNAs increased in *pnp1-1* at least slightly (*trnQ*) or markedly (*trnT.1* and *trnS.3*). In addition to tRNA regulation, cpPNPase has been implicated in the degradation of group II introns following splicing (Germain *et al.* 2011). We observed here that the *rps16* intron overaccumulates in *pnp1-1* as multiple larger transcripts and a single smaller transcript, in addition to the WT transcripts. The increase in *rps16* intron abundance correlated with an increase in abundance of as-*rps16*i-1, which is barely detectable in the WT. An analogous positive correlation was observed for *psbB*.

An opposite phenomenon was observed for nc-*rbcL-accD*int and the corresponding sense strand intergenic region. In this case, a smaller and less abundant ncRNA correlated with accumulation of the complementary region, which does not accumulate in the WT. Another inverse correlation was observed for the *ndhF* 5′ end. Although these correlations are intriguing, and a causal relationship would be consistent with known functions of ncRNAs in prokaryotes, the behaviors of complementary transcripts could also be independent effects of cpPNPase depletion. Additional experimentation will be required to make this determination.

### Chloroplast ncRNAs exist as primary transcripts and processed species, and are transcribed by two RNA polymerase types

Chloroplast genes can be transcribed from their own promoter and/or cotranscribed with an upstream gene by either a nuclear-encoded phage-like RNA polymerase (NEP) or the bacterial-like PEP (Liere and Borner 2008). PEP-regulated genes often encode proteins involved in photosynthesis, whereas many NEP-regulated genes maintain gene expression functions. Additionally, PEP- and NEP-regulated genes are expressed differentially throughout plastid development (Liere and Maliga 2001). We elected to examine several ncRNAs from this viewpoint to begin to define ncRNA biogenesis pathways in the chloroplast.

**Figure 4:**
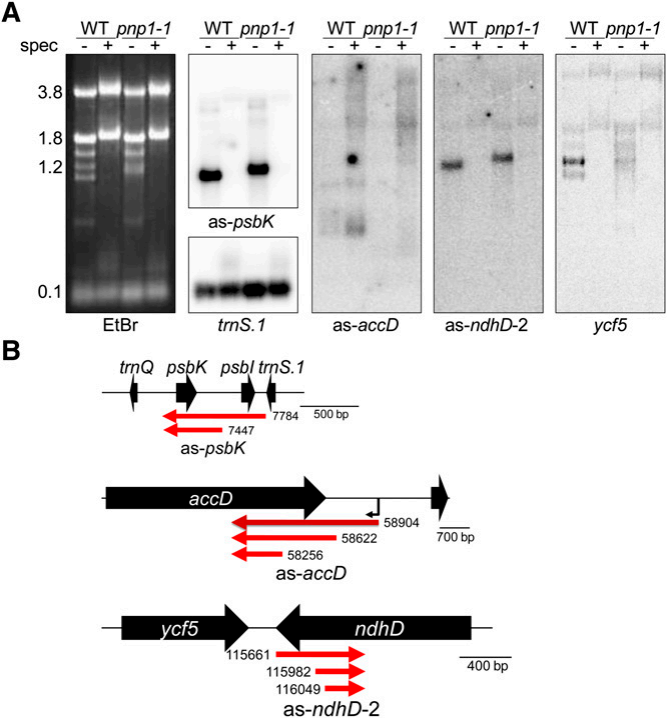
Promoter analysis of three ncRNAs. (A) RNA blots of ncRNAs from wild-type (WT) and *pnp1-1* RNA from plants grown with (+) and without (−) spectinomycin (spec). Probes are indicated below each panel, and an ethidium bromide (EtBr)–stained gel is shown with rRNA sizes (nt; left). The *trnS.1* and *ycf5* sense RNAs were hybridized with a double-stranded DNA probe. (B) Confirmed ncRNA gene models showing the major 5′ ends from processed (red arrows) and primary (dark red arrow) transcripts mapped using RACE +/− TAP treatment. A putative promoter (bent arrow) is shown for as-*accD*.

Transcription by PEP *vs.* NEP was determined by germinating plants on media with and without spectinomycin. Spectinomycin inhibits plastid translation and, therefore, PEP synthesis, creating chlorophyll-deficient plants with only NEP activity (Swiatecka-Hagenbruch *et al.* 2007). Confirmation of PEP inhibition can be seen from the ethidium bromide–stained gel in which the plastid rRNAs are greatly reduced or absent (Figure 4A, left panel). Analysis of these samples by RNA gel blot revealed as-*psbK* and as-*ndhD*-2 to be PEP dependent, whereas as-*accD* is transcribed by NEP. For the two PEP-dependent ncRNAs, upstream genes on the same strand could give rise to the ncRNAs by read-through transcription. We found, however, that *trnS.1* is PEP independent, unlike the downstream ncRNA. On the other hand, *ycf5* and as-*ndhD*-2 were both PEP dependent, consistent with a cotranscription model.

To ascertain whether these ncRNAs have dedicated promoters, their 5′ ends were mapped using 5′ RACE with or without prior treatment by tobacco acid phosphatase (TAP; Figure S2). Chloroplast primary transcripts are triphosphorylated and amenable to RNA ligase–mediated oligonucleotide addition only after TAP treatment removes the 5′ diphosphate (Bensing *et al.* 1996). Conversely, processed transcripts do not require TAP treatment for oligonucleotide adaptation. RACE analysis revealed two 5′ ends for as-*psbK*, as diagrammed in Figure 4B, that are not TAP dependent. The longer transcript has its 5′ end precisely at the 3′ end of the upstream *trnS.1* coding region (Figure S2, band a), which suggests that the asRNA 5′ end is derived from 3′ processing of a tRNA precursor. The second, shorter as-*psbK* transcript (Figure S2, band b), with its end between *psbK* and *psbI*, is likely a processing product of the larger species.

Analysis of as-*accD* revealed three 5′ ends, the longest of which was TAP dependent (Figure S2, band c), indicating that it is a primary transcript. The region upstream of as-*accD* is AT-rich and includes a putative type-1a YRTA NEP consensus motif (TATA) at -8, consistent with expression on spectinomycin media (Hajdukiewicz *et al.* 1997; Swiatecka-Hagenbruch *et al.* 2007). Given that there is no immediate upstream gene transcribed in the same direction, the presence of an ncRNA-specific promoter was not unexpected. Lastly, three as-*ndhD*-2 5′ ends were mapped, none of which were TAP dependent (Figure S2, bands f–h), consistent with the cotranscription model proposed above. The longest transcript extended just past the 3′ end of the complementary *ndhD* gene, whereas the other two 5′ ends were internal to the *ndhD* coding region.

### Depletion of chloroplast exo- and endoribonucleases differentially affects ncRNA maturation and accumulation

**Figure 5:**
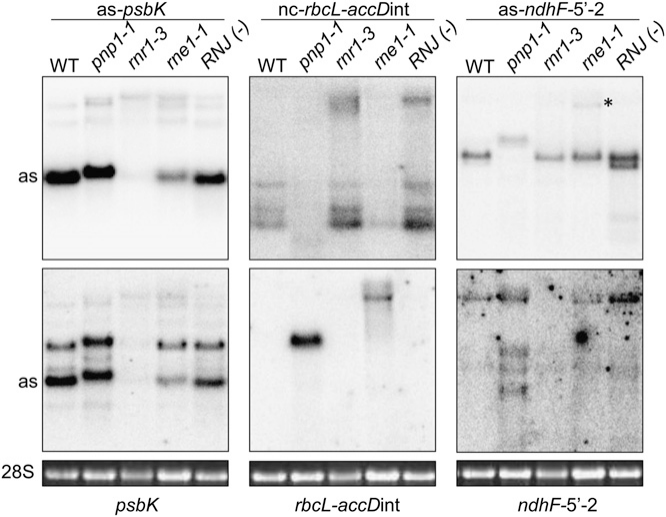
Analysis of three ncRNAs (top panel) and the complementary transcripts (lower panel) in chloroplast ribonuclease-deficient samples. The 28S rRNA was ethidium bromide stained to reflect loading (bottom). Null mutants for PNPase (*pnp1-1*), RNase R (*rnr1-3*), and RNase E (*rne1-1*), and VIGS knockdown tissue for RNase J [RNJ(−)] were used. Blots were analyzed as described in Figure 3. The as-*psbK* blot was reprobed to detect the sense transcript (the as-*psbK* species is marked “as” in both panels). An as-*ndhF*5′-2 precursor that accumulates in *rne1-1* is marked by an asterisk.

In bacteria, the ribonucleases PNPase, RNase E, and RNase III have been shown to regulate ncRNA stability (Viegas *et al.* 2007; Andrade and Arraiano 2008; Stead *et al.* 2010). Therefore, we examined the roles of chloroplast ribonucleases in ncRNA accumulation and biogenesis (Figure 5). These were chloroplast PNPase, RNase R, a 3′→5′ hydrolytic exoribonuclease; RNase E, an endoribonuclease; and RNase J, whose known activities in bacteria include 5′→3′ exoribonuclease and endoribonuclease (Deikus *et al.* 2008). Published studies for chloroplasts have implicated RNase R in rRNA maturation (Bollenbach *et al.* 2005) and regulation of the asRNA AS5 (Sharwood *et al.* 2011), and RNase E in polycistronic RNA cleavage (Walter *et al.* 2010). Although null mutants for RNase R and RNase E are viable, RNase J deficiency is embryo-lethal, so we used tissue partly depleted for the enzyme by VIGS (Sharwood *et al.* 2011).

The left column of Figure 5 shows analysis of as-*psbK* and *psbK*. In the absence of RNase R, neither as-*psbK* nor *psbK* was detected, and in the absence of RNase E, both transcripts underaccumulated. Thus, there is a positive correlation between *psbK* and as-*psbK* accumulation. One possibility is that RNase R and RNase E are part of a maturation pathway for *psbK* and/or as-*psbK*, and in their absence, misprocessed transcripts may be readily degraded.

The center column of Figure 5 shows nc-*rbcL-accD*int and the corresponding sense intergenic region. We found that nc-*rbcL-accD*int underaccumulates in *pnp1-1*, as shown in Figure 3, and in *rne1-1*. In both cases, underaccumulation of the ncRNA correlated with accumulation of the sense strand. As *rbcL* and *accD* are transcribed from individual PEP and NEP promoters, respectively, the intergenic region detected is likely an extension of an *rbcL* precursor rather than an unprocessed dicistron (Gruissem and Zurawski 1985; Hirata *et al.* 2004). PNPase and RNase E appear to have a role in *rbcL* maturation that leads to degradation of the intergenic region, as this transcript is not detectable in the WT. We also noted a larger form of nc-*rbcL-accD*int in *rnr1-3* and RNJ(−), implying that these ribonucleases are partially involved in the biogenesis of this ncRNA.

Finally, we analyzed the 5′ end of the *ndhF* gene (Figure 5, right column). The asRNA was present as a doublet in WT, which was slightly longer in *pnp1-1*, likely due to a 3′ extension. In *rne1-1*, a much longer form was detected (asterisk) and the lower doublet band was absent. This lower band was also absent from *rnr1-3*. In RNJ(−), however, as-*ndhF*-5′-2 accumulated in the same forms as in the WT, although the abundance was increased. Together these results indicate that this asRNA undergoes posttranscriptional processing to the mature forms found in the WT. Aberrant as-*ndhF-*5′-2 processing coincided with altered *ndhF* sense transcript processing and abundance. In the WT, *ndhF* accumulated as a single transcript, whereas in *pnp1-1*, this transcript was apparent, along with three smaller transcripts. The sense transcript was reduced in both *rnr1-3* and *rne1-1*, whereas it overaccumulated in RNJ(−). It remains to be determined whether the correlations between the sense and antisense transcripts are due to direct interactions. However, the data presented reveal roles for many enzymes in determining the accumulation and form of chloroplast ncRNAs, which likely reflects a variety of biogenesis pathways.

## Discussion

Knowledge of the extent and function of ncRNAs in prokaryotes and eukaryotes has grown considerably over the last 20 years, fueled by advances in sequencing technology and bioinformatics. However, information on the occurrence of ncRNAs in organelles, specifically the chloroplast, has been limited in scope and depth (Marker *et al.* 2002; Lung *et al.* 2006; Georg *et al.* 2010; Hotto *et al.* 2010; Mohorianu *et al.* 2011; Wang *et al.* 2011; Zghidi-Abouzid *et al.* 2011). Here, we employed high-throughput, strand-specific RNA sequencing (Lister *et al.* 2008) to identify the bulk of stably expressed *Arabidopsis* chloroplast ncRNAs >100 nt in length. These ncRNAs are transcribed by at least two RNA polymerase types, and their accumulation is affected by a variety of chloroplast ribonucleases. It is likely that some proportion of them plays a role in chloroplast gene expression through pathways that are largely undefined.

### Identification of chloroplast ncRNAs using RNA-Seq

Two recent studies using RNA-Seq identified small chloroplast RNAs in cabbage (Wang *et al.* 2011) and tomato fruit (Mohorianu *et al.* 2011), excluding transcripts >100 nt. Other studies have used cDNA library sequencing and computational approaches to identify chloroplast ncRNAs (Lung *et al.* 2006; Georg *et al.* 2010), and small numbers of polyadenylated ncRNAs are present in EST libraries. However, there are several limitations to these methods, including low sensitivity, deliberate length restrictions, and reliance on specific sequence characteristics.

The RNA-Seq method used here has a large dynamic range, facilitating discovery of low abundance transcripts (Wang *et al.* 2009), and it identifies expressed transcripts rather than relying on predictions of conserved sequence or structural features (Pichon and Felden 2008). This is important for chloroplast ncRNA identification, as promoters often share little sequence homology and inefficient transcription termination can result in multiple 3′ ends (Bollenbach *et al.* 2008; Liere and Borner 2008). At the same time, this RNA-Seq method is restricted by strong RNA secondary structures, which can reduce the efficiency at which some oligonucleotides ligate to the oligonucleotides 5′ and 3′ ends. This drawback is mitigated by fractionation of the total RNA pool prior to oligonucleotide adaptation. Also, our method largely excludes RNAs of <80 nt, a tradeoff that reduces the amount of tRNAs and small rRNAs that would otherwise be highly sequenced. The pool of <80 nt chloroplast ncRNAs, however, has been covered by other studies cited above.

**Figure 6:**
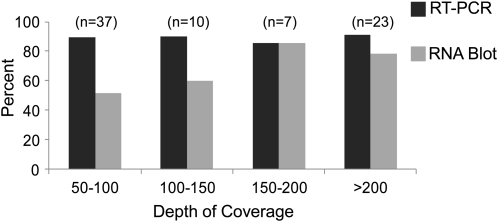
Percentage of ncRNAs detected by either RT-PCR (black bars) or RNA gel blot (gray bars) compared with the depth of RNA-Seq coverage. Total number of ncRNAs in each category prior to verification is in parentheses.

Given that RNA-Seq yielded over 10 million sequences that aligned to the chloroplast genome from each genotype, it was important to filter this dataset for our purposes. Location was primarily constrained to regions antisense to known coding sequences to enrich for possible *cis*-encoded ncRNAs, and a threshold of ≥50× coverage per transcript was used to trigger follow-up analysis. Indeed, when the coverage of a potential ncRNA reached 50–100×, 50% were confirmed by gel blot, and nearly 90% by RT-PCR, whereas >80% could be confirmed by gel blot when >150× coverage was obtained (Figure 6). Given these outcomes, a lower threshold (*e.g.* 25–50× coverage) may identify a certain number of additional low abundance ncRNAs (*i.e.* those detectable by RT-PCR but less readily detectable by gel blot). For instance, a formerly characterized chloroplast asRNA, AS5, did not meet the RNA-Seq criteria due to its low endogenous expression (Hotto *et al.* 2010; Sharwood *et al.* 2011). Additionally, some chloroplast ncRNAs may only be expressed under particular developmental or stress conditions that were not used in this study.

Although we have called this RNA population ncRNAs, it is inevitable that some will have small open reading frames (ORF), either fortuitous or functional. In two cases, conserved open reading frames were present. The ncRNA as-*psbK* includes a 49 amino acid ORF with 72% identity to ORF44, which is encoded in an analogous position and stably transcribed in barley chloroplasts (Sexton *et al.* 1990) although it has no known function. A second example is a 58 amino acid ORF found within as-*ndhD*, which has 75–98% identity to putative proteins encoded by the *ndhD* antisense strand in numerous chloroplast genomes. Although this ORF also has no attributed function, the apparent evolutionary selection for ORFs on both strands is striking.

### ncRNAs are encoded throughout the plastome

**Figure 7:**
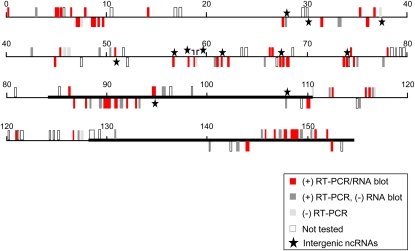
Summary of ncRNA analysis. The chloroplast genome is depicted in linear form, with the inverted repeats shown as heavy lines. The zero position corresponds to the beginning of the large single-copy region. Each ncRNA tentatively identified by RNA-Seq is shown; the legend at bottom right indicates the verification status.

At a coverage of at least 1× per nt, RNA-Seq data from both the WT and *pnp1-1* spanned >99% of both chloroplast genome strands. From this, 107 putative ncRNAs met filtering criteria, and their distribution and verification status are shown in Figure 7. Of these, only as-*rps16*-I was identified in a previous study (Lung *et al.* 2006). Full symmetric transcription of the chloroplast genome is consistent with its well-described inefficient transcription termination, and it implies a heavy reliance on posttranscriptional regulatory mechanisms. Unwanted transcripts are likely distinguished from functional ones by the collective effects of RNA structures, RNA-binding proteins, and their sensitivities to RNases. We speculate that nonfunctional or detrimental ncRNAs are rapidly degraded, because *cis*-encoded asRNAs are inherently inhibitory to gene expression, a view supported by three published examples for chloroplasts (Nishimura *et al.* 2004; Hegeman *et al.* 2005; Hotto *et al.* 2010). This implies the ncRNAs accumulating to detectable levels may well retain functional roles.

Chloroplast ncRNAs include both primary and processed transcripts emanating from ncRNA-specific promoters (*e.g.* as-*accD*; Figure 4) and those generated by cotranscription with an upstream gene. For example, as-*ndhD*-2 appears to be cotranscribed with *ycf5*, and as-*psbK* with *trnS.1* (Figure 4B). There is a discrepancy with as-*psbK* because it fails to accumulate when plants are grown on spectinomycin (Figure 4A), showing it requires PEP, whereas the upstream *trnS.1* gene is transcribed by NEP (Figure 4; Gruissem *et al.* 1986; Wu *et al.* 1997). We suggest that as-*psbK* is transcribed from an unidentified PEP promoter upstream of *trnS.1*, whereas initiation at its NEP promoter leads to transcription termination without read-through into as-*psbK*. In support of this hypothesis, we note that the as-*psbK* probe identified a 3.7 kb transcript that may extend upstream of *trnS.1* and is PEP dependent (Figures 3A and 4A).

### Chloroplast ncRNA maturation and accumulation are affected by ribonucleases

We assessed the effects on ncRNA biogenesis of deficiencies for the two known chloroplast 3′→5′ exoribonucleases, cpPNPase and RNase R, the endoribonuclease RNase E, and the putative endoribonuclease and 5′→3′ exoribonuclease RNase J. The results were diverse, suggesting that there are several pathways responsible for ncRNA processing and accumulation.

PNPase was previously shown to regulate bacterial ncRNAs, and the eukaryotic exosome (similar in function to PNPase) assists in the maturation and degradation of nuclear ncRNAs (Houseley *et al.* 2006; Viegas *et al.* 2007). Because cpPNPase plays a broad role in RNA metabolism (Germain *et al.* 2011), it was not surprising that it also acts on ncRNAs. Our data suggest two functions, namely, modulation of ncRNA abundance and 3′ end maturation.

Loss of RNase R had a major effect on both sense and antisense *psbK* RNAs; both were nearly depleted in mutant tissue. Chloroplast RNase R has previously been implicated in RNA maturation and accumulation of the asRNA AS5, transcribed from the rDNA region (Sharwood *et al.* 2011). In fact, *rnr1-3* accumulates only 6% of the WT level of 5S rRNA, giving precedent for reduced RNA accumulation in this mutant. In that case, however, the asRNA increases in abundance, which led to the hypothesis that the asRNA destabilizes the sense transcript. In the case of *psbK*, the transcripts are coordinately affected, raising the possibility that they protect one another. The proximal cause of instability of one or both in the absence of RNase R remains to be ascertained.

RNase E deficiency appeared to have a destabilizing effect on as-*psbK*, *psbK*, and nc-*rbcL-accD*int, whereas the *rbcL-accD*int sense strand transcript increased in abundance. Some *E. coli* ncRNAs were destabilized in the absence of RNase E, which may be due to direct RNase E–ncRNA interactions or may result from changes in other mRNAs or unidentified interactors (Stead *et al.* 2010). RNase E could have a similar role with respect to chloroplast ncRNAs. The appearance of a sense strand *rbcL-accD* intergenic region is consistent with the postulated role of chloroplast RNase E in polycistronic transcript cleavage (Walter *et al.* 2010). Whether this transcript somehow destabilizes its antisense counterpart remains to be determined.

RNase J has been little studied in chloroplasts. RNase E and RNase J have been shown to be partially redundant in *Arabidopsis* 5S-*trnR* processing, and RNase J has been shown to replace some RNase E functions in *B. subtilis* (Britton *et al.* 2007; Sharwood *et al.* 2011). We found here that RNase J–deficient material accumulated an nc-*rbcL-accD*int precursor and had minor quantitative alterations for other ncRNAs. Taken together, these results indicate that multiple ribonucleases are involved in chloroplast ncRNA maturation and degradation.

### Possible functions of chloroplast ncRNAs

The ncRNAs identified in this study had origins throughout the chloroplast genome (Figure 7). Because accumulating asRNAs are complementary to all types of coding regions, including tRNAs, photosynthetic genes, and gene expression machinery, their regulatory roles could be quite diverse, similar to large-scale mechanisms of gene regulation attributed to bacterial ncRNAs (Repoila and Darfeuille 2009). The focus of this study was on *cis*-encoded asRNAs, although we cannot exclude the possibility that one or more also acts in *trans*. Potential functions of *cis*-encoded asRNAs are suggested by the region of complementarity to the corresponding sense RNA, as well as by the target mRNA location within an operon.

At least 31 chloroplast ncRNAs overlap the translation initiation region of a complementary mRNA. In bacteria, base pairing of ncRNAs to the 5′ end of target transcripts is common. The outcome can be activation or repression of translation by altering ribosome accessibility to the Shine-Dalgarno (SD) sequence and/or start codon (Regnier and Hajnsdorf 2009). However, chloroplast mRNAs frequently lack SD elements and instead require upstream *cis*-elements and gene-specific *trans*-factors (Peled-Zehavi and Danon 2007), which may be targets of ncRNAs. The *in vitro* translation system available for chloroplasts offers one avenue to test these hypotheses (Yukawa *et al.* 2007).

At least 39 chloroplast ncRNAs are complementary to the sense strand mRNA 3′ end. Bacterial ncRNAs that bind to mRNA 3′ ends often stabilize the mRNA by blocking 3′→5′ exoribonucleases. This is the case for *E. coli* GadY, which stabilizes the *cis*-encoded GadX mRNA (Opdyke *et al.* 2004). Chloroplast transcript 3′ ends are often stabilized by a stem-loop–forming inverted repeat, but not all mRNAs possess predicted 3′ stem loops, and these would be candidates for ncRNA-mediated stabilization. A possible example is as-*psbT*, which was hypothesized to stabilize the complementary *psbT* mRNA under oxidative stress conditions (Zghidi-Abouzid *et al.* 2011).

Approximately 40 chloroplast asRNAs are complementary only to the coding region of mRNAs, and some of these putative mRNA targets are within operons. Bacterial ncRNAs that bind to their target within the coding region can alter transcript stability by creating or blocking a ribonuclease binding site (Pfeiffer *et al.* 2009). Additionally, bacterial ncRNAs can alter transcript accumulation within an operon, one example being RhyB, which downregulates the *iscSUA* genes within the *iscRSUA* operon to allow independent accumulation of *iscR* (Desnoyers *et al.* 2009). Similar scenarios could be envisioned for chloroplast ncRNAs, leading to differential accumulation of transcripts within a gene cluster.

Overall, RNA-Seq analysis proved extremely useful for identifying chloroplast ncRNAs, as approximately 45% were further validated by experimental means. We suspect that this method will also be useful for characterization of other transcriptomes, particularly organellar ones that benefit from the deep coverage of the low-complexity genomes inherent in total RNA analysis.

## Supplementary Material

Supporting Information
