# Supplementary material for: Unexpected Diversity of Chloroplast Noncoding RNAs as Revealed by Deep Sequencing of the Arabidopsis Transcriptome
Source: G3 (Bethesda). 2011 Dec 1;1(7):559–70. doi: 10.1534/g3.111.000752 (PMC3276175; doi:10.1534/g3.111.000752)
Supplement: Supporting Information [file supp_1_7_559__index.html]

Supporting Information 

# Unexpected Diversity of Chloroplast Noncoding RNAs as Revealed by Deep Sequencing of the *Arabidopsis* Transcriptome

## Supporting Information for Hotto *et al.*, 2011

**Files in this Data Supplement:**

- Supporting Information - Figures S1 and S2 and Tables S1 and S2 (PDF, 2 MB)
- Figure S1 - RNA blots of ncRNAs identified through strand-specific sequencing (PDF, 1.8 MB)
- Figure S2 - Analysis of ncRNA 5� ends (PDF, 168 KB)
- Table S1 - Primers (PDF, 24 KB)
- Table S2 - Both sense and antisense reads were binned into chloroplast genes (from annotated transcription start site to end site) (PDF, 36 KB)
