# Supplementary material for: Unexpected Diversity of Chloroplast Noncoding RNAs as Revealed by Deep Sequencing of the Arabidopsis Transcriptome
Source: G3 (Bethesda). 2011 Dec 1;1(7):559–70. doi: 10.1534/g3.111.000752 (PMC3276175; doi:10.1534/g3.111.000752)
Supplement: Supporting Information [file supp_1.7.559_000752SI.pdf]

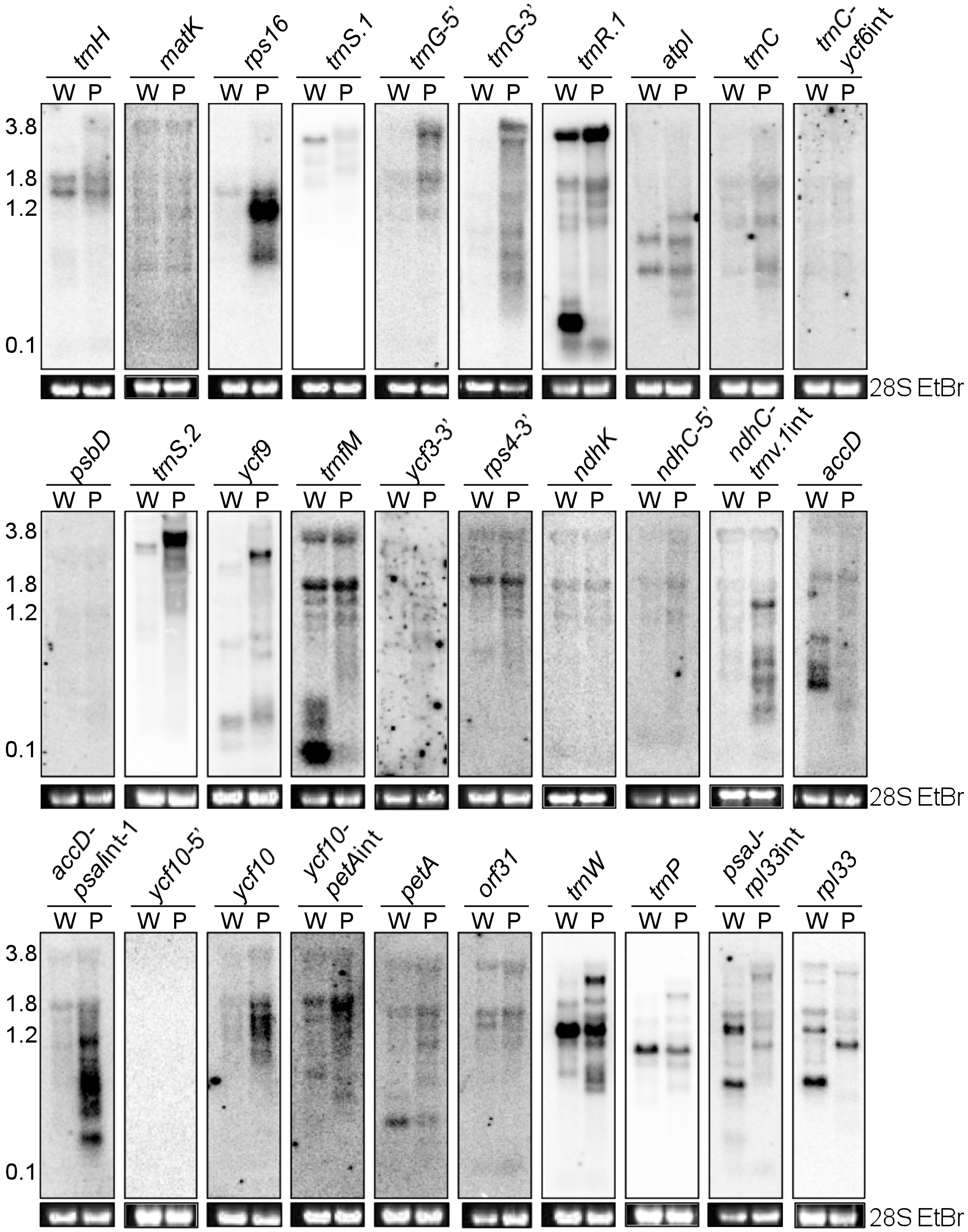

Figure S1 RNA blots of ncRNAs identified through strand-specific sequencing. Details of each ncRNA are in Table 1. Samples were loaded in the order: wild-type (W) and *pnp1-1* (P). Sizes of rRNAs (nt) are shown at the left.

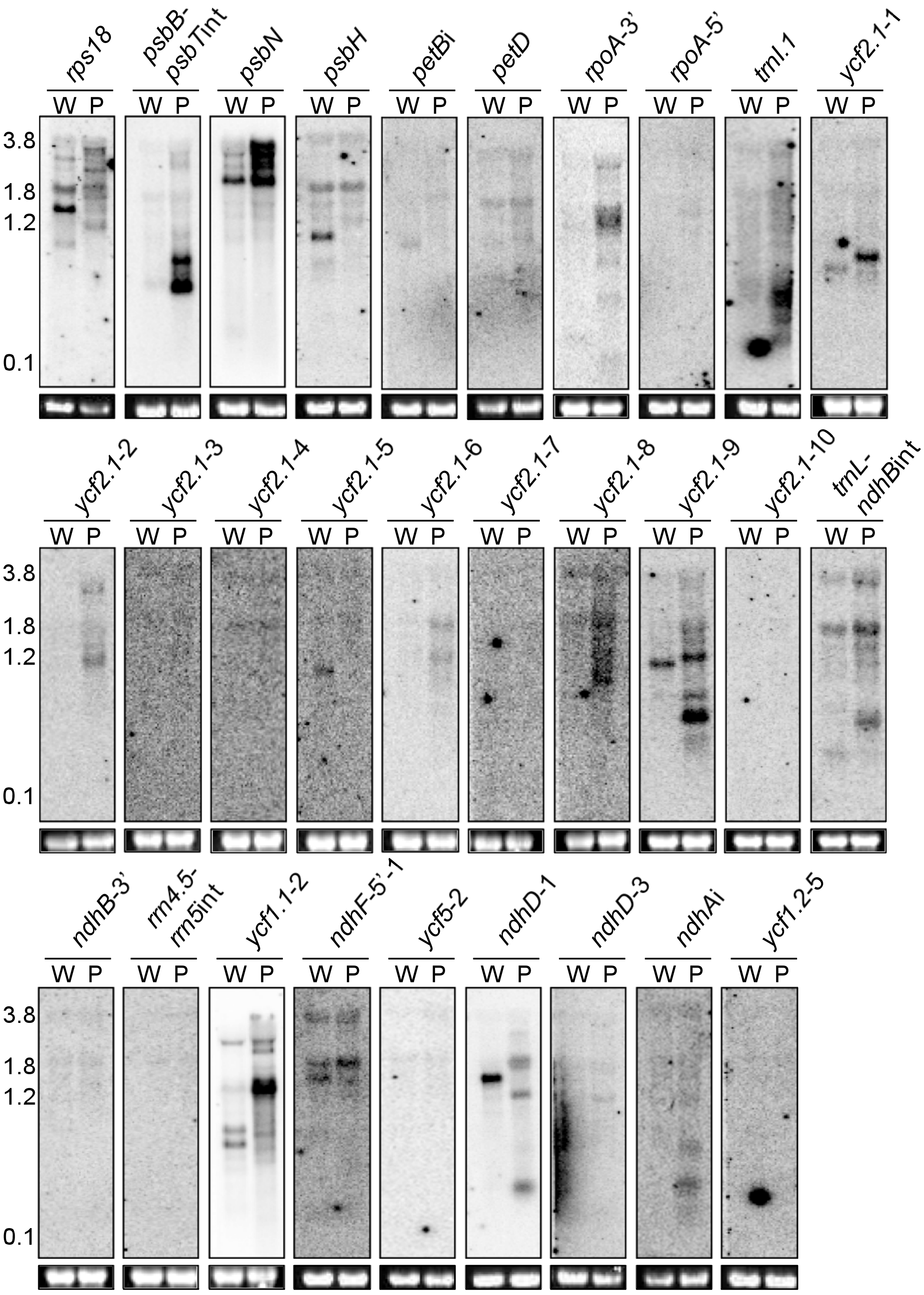

Figure S1 cont. RNA blots of ncRNAs identified through strand-specific sequencing. Details of each ncRNA are in Table 1. Samples were loaded in the order: wild-type (W) and *pnp1-1* (P). Sizes of rRNAs (nt) are shown at the left.

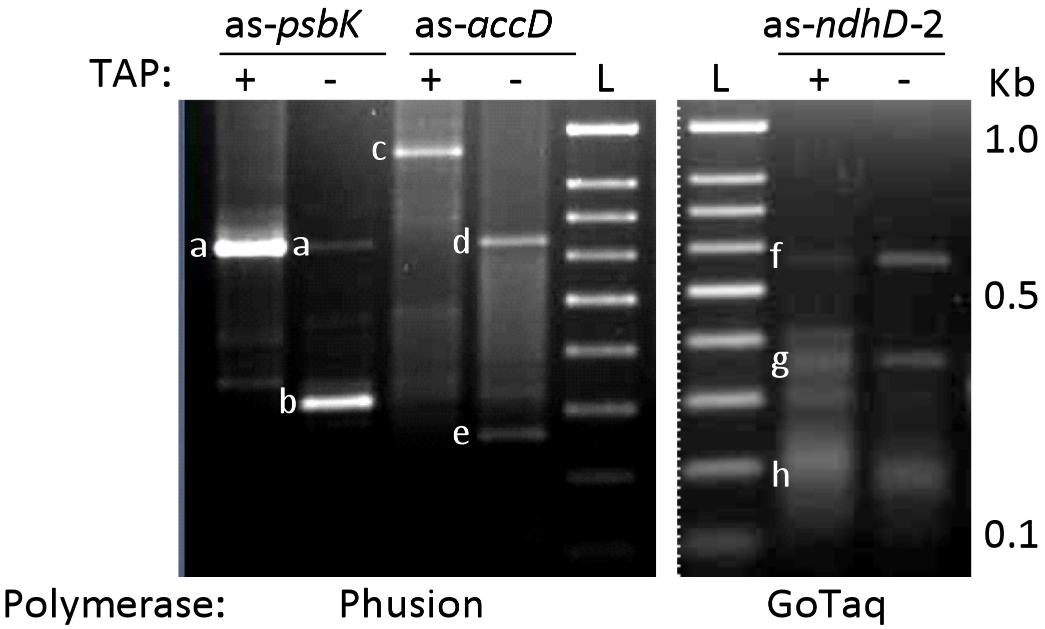

Figure S2 Analysis of ncRNA 5' ends. The 5' ends of ncRNAs were analyzed with and without treatment by Tobacco Acid Phosphatase (TAP) using RACE. The cDNA ends were amplified using nested PCR by the DNA polymerase indicated below the gel. Major transcripts that were sequenced are indicated with letters (a-h). A 100 bp DNA ladder (L) was loaded as a size reference, and sizes (kb) are indicated at the right. Samples were separated in a 1% agarose gel stained with ethidium bromide.

Tables S1 and S2

Tables S1 and S2 are available for download at http://www.g3journal.org/lookup/suppl/doi:10.1534/g3.111.000752/-/DC1 as Excel files.

Table S1 Primers

Table S2 Both sense and antisense reads were binned into chloroplast genes (from annotated transcription start site to end site).
