# Supplementary material for: Unexpected Diversity of Chloroplast Noncoding RNAs as Revealed by Deep Sequencing of the Arabidopsis Transcriptome
Source: G3 (Bethesda). 2011 Dec 1;1(7):559–70. doi: 10.1534/g3.111.000752 (PMC3276175; doi:10.1534/g3.111.000752)
Supplement: Supporting Information [file supp_1.7.559_FigureS1.pdf]

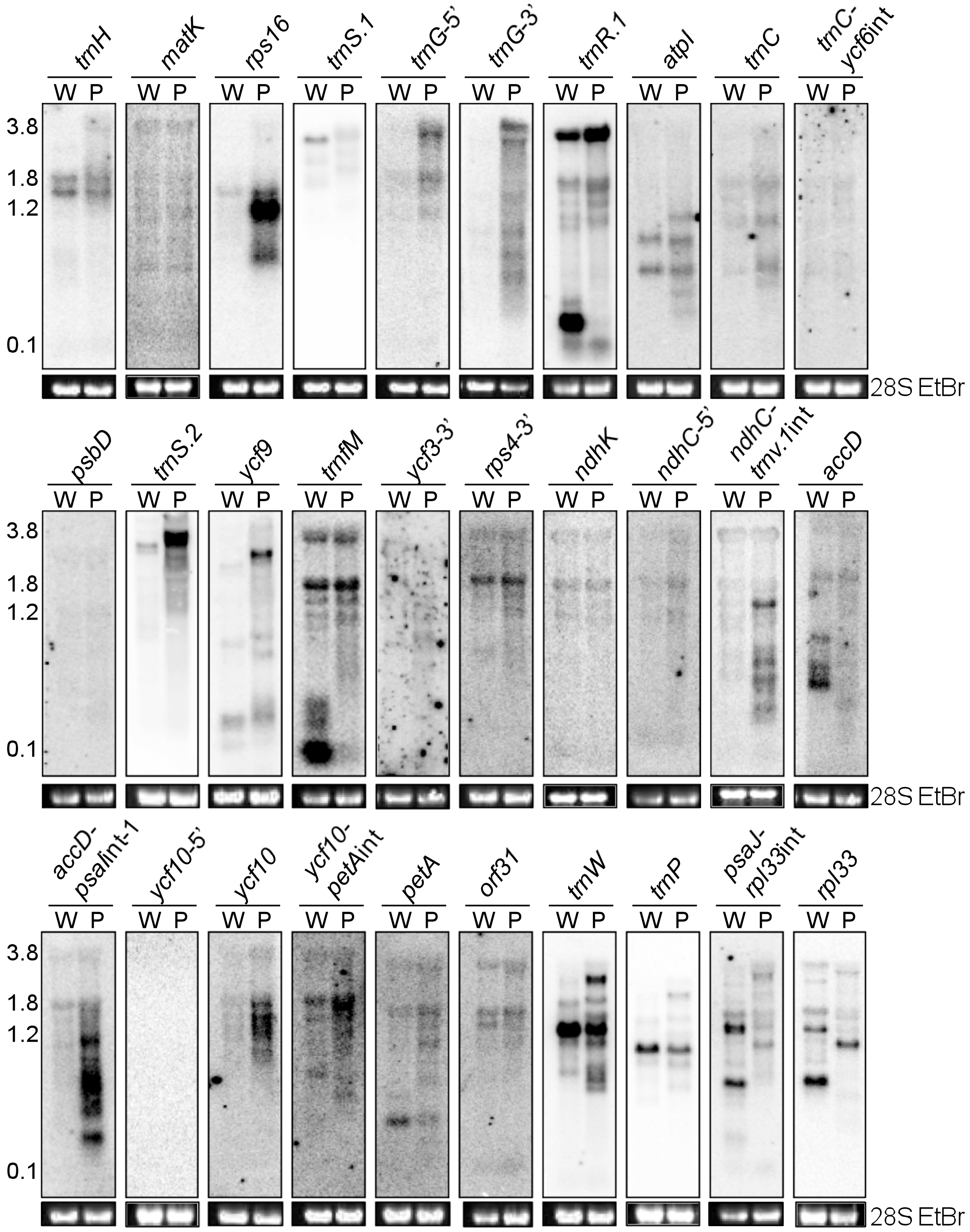

Figure S1 RNA blots of ncRNAs identified through strand-specific sequencing. Details of each ncRNA are in Table 1. Samples were loaded in the order: wild-type (W) and *pnp1-1* (P). Sizes of rRNAs (nt) are shown at the left.

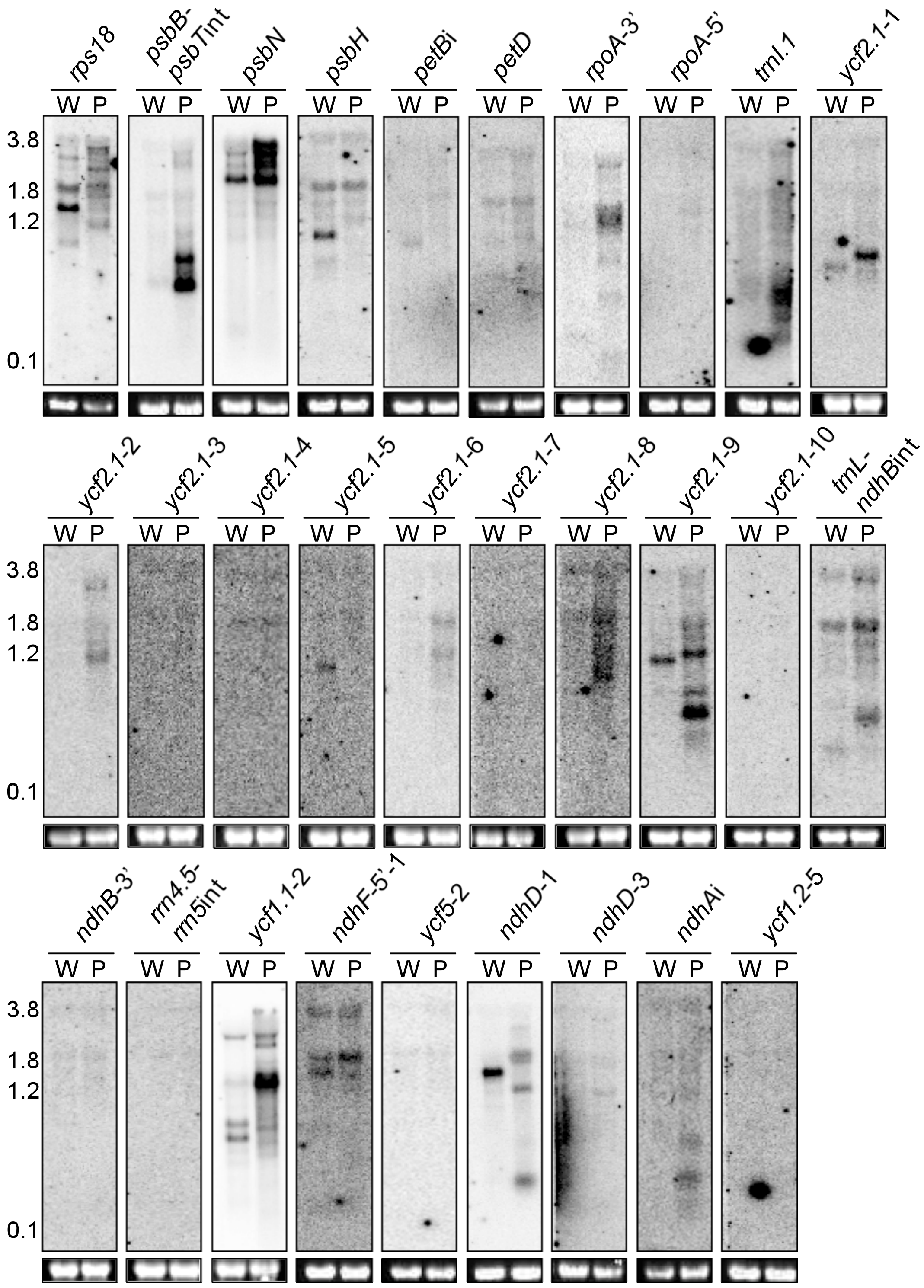

Figure S1 cont. RNA blots of ncRNAs identified through strand-specific sequencing. Details of each ncRNA are in Table 1. Samples were loaded in the order: wild-type (W) and *pnp1-1* (P). Sizes of rRNAs (nt) are shown at the left.
