# Supplementary material for: Unexpected Diversity of Chloroplast Noncoding RNAs as Revealed by Deep Sequencing of the Arabidopsis Transcriptome
Source: G3 (Bethesda). 2011 Dec 1;1(7):559–70. doi: 10.1534/g3.111.000752 (PMC3276175; doi:10.1534/g3.111.000752)
Supplement: Supporting Information [file supp_1.7.559_FigureS2.pdf]

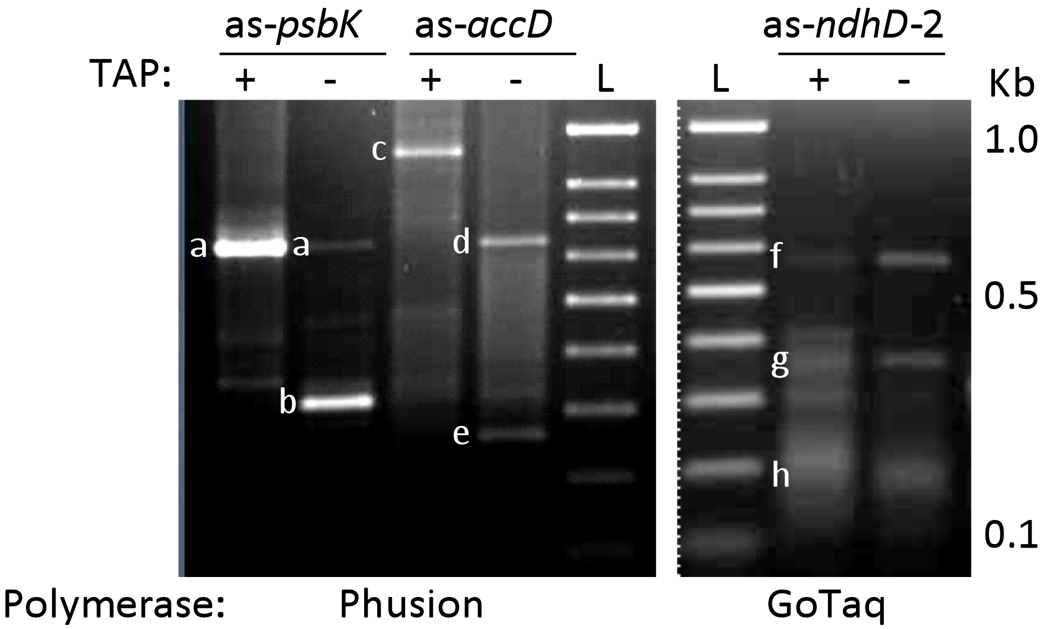

Figure S2 Analysis of ncRNA 5' ends. The 5' ends of ncRNAs were analyzed with and without treatment by Tobacco Acid Phosphatase (TAP) using RACE. The cDNA ends were amplified using nested PCR by the DNA polymerase indicated below the gel. Major transcripts that were sequenced are indicated with letters (a-h). A 100 bp DNA ladder (L) was loaded as a size reference, and sizes (kb) are indicated at the right. Samples were separated in a 1% agarose gel stained with ethidium bromide.
